# Transient increase of ATP as a response to temperature up-shift in *Escherichia coli*

**DOI:** 10.1186/1475-2859-4-9

**Published:** 2005-04-01

**Authors:** Jaakko Soini, Christina Falschlehner, Christina Mayer, Daniela Böhm, Stefan Weinel, Johanna Panula, Antti Vasala, Peter Neubauer

**Affiliations:** 1 Bioprocess Engineering Laboratory and Biocenter Oulu, Department of Process and Environmental Engineering, University of Oulu, P.O.Box 4300, FI – 90014 Oulu, Finland; 2 Institute for Biotechnology, Department of Biochemistry/Biotechnology, Martin-Luther-University Halle-Wittenberg, Kurt-Mothes-Str. 3, D-06120 Halle, Germany

## Abstract

**Background:**

*Escherichia coli *induces the heat shock response to a temperature up-shift which is connected to the synthesis of a characteristic set of proteins, including ATP dependent chaperones and proteases. Therefore the balance of the nucleotide pool is important for the adaptation and continuous function of the cell. Whereas it has been observed in eukaryotic cells, that the ATP level immediately decreased after the temperature shift, no data are available for *E. coli *about the adenosine nucleotide levels during the narrow time range of minutes after a temperature up-shift.

**Results:**

The current study shows that a temperature up-shift is followed by a very fast significant transient increase of the cellular ATP concentration within the first minutes. This increase is connected to a longer lasting elevation of the cellular respiration and glucose uptake. Also the mRNA level of typical heat shock genes increases within only one minute after the heat-shock.

**Conclusion:**

The presented data prove the very fast response of *E. coli *to a heat-shock and that the initial response includes the increase of the ATP pool which is important to fulfil the need of the cell for new syntheses, as well as for the function of chaperones and proteases.

## Background

Bacteria, as all kind of organisms, respond to a sudden increase of the temperature by a heat shock response which induces a specific set of proteins. This stress system belongs to the best-studied cellular responses. As the protein composition of a cell determines substantially the cellular functions, the heat shock response is mainly understood in terms of protein synthesis and composition.

The heat shock response of *E. coli *is mediated by the alternative sigma factor σ^32 ^(*rpoH *gene product) and leads to induction of more than 20 heat shock proteins [1,2]. In difference to other stress responses of *E. coli *which in a regulatory cascade first lead to synthesis of the respective sigma factors which then, consecutively, direct the transcription of their genes, the transcriptional induction of the heat shock proteins can start very fast after a heat shock. This first fast synthesis of heat shock proteins is transient, followed by an adaptation period with a lower rate of heat shock protein synthesis and later reaches a new steady-state level [1,3]. This is because a cell also under non-stress conditions contains a set of a low number of σ^32 ^molecules (about 10–30 molecules; [4,5]). Those molecules although possessing a high binding affinity for the RNA polymerase [6], however, are rarely transcriptionally active, because of sequestering by binding to DnaK, which together with its cochaperones DnaJ and GrpE associates with σ^32 ^[7,8] and mediates its degradation by the FtsH protease [9]. The release of σ^32 ^from the DnaK chaperone is regulated by the amount of missfolded proteins which may accumulate either when existing proteins lose their structure, or when new-synthesised proteins do not fold properly, which is the characteristic intracellular signal of a heat shock [10] but also may occur under strong overexpression of recombinant proteins [11]. Once released from DnaK, σ^32 ^binds to RNA polymerase, is stabilised and initiates the synthesis of the heat shock proteins. This mechanism due to the high affinity of σ^32 ^for binding to RNA polymerase results in the synthesis of heat shock proteins to a concentration of about 5 % of the total cellular protein [1].

The physiological response of *E. coli *towards a heat shock is more complex than just the synthesis of heat shock proteins, including transient growth stop, changes in the cell membranes due to changes in the ratio of lipids and integral membrane proteins [12], and probably also transient DNA relaxation [13]. In higher eukaryotes, one of the most immediate effects of a heat shock is an extensive disruption of the cytoskeleton [14,15]. From the metabolic side already early studies have shown that the maintenance energy requirement increases with increasing growth temperature [16-19] and the yield coefficient Y_X/S _was observed to decrease, possibly in connection to the higher protein turnover [20]. A number of authors also reported increased respiration rates in relation to the specific growth rate at higher temperatures, e.g. for *A. aerogenes *(*Klebsiella pneumoniae*) [21]. (referred by [22])

The group of more than 30 heat shock proteins involves mainly proteases and chaperones, being involved in the degradation of improper folded proteins. The function of these proteins is connected to the hydrolysis of ATP, such as proteolysis by Lon, FtsH and Clp in *E. coli *[23-25], and the action of the chaperone complexes GroEL/ES and DnaK/J/GrpE [26,27]. Although a number of *in vitro *studies have been performed revealing this ATP dependence (e.g. [28]), only limited information is available about the response of the *in vivo *ATP level after a temperature shift. Different authors (e.g. [29-31]) found in higher eukaryotes a fast decrease of the ATP level to less than 50 % after a temperature up-shift parallel to induction of heat shock proteins.

In *E. coli *the ATP concentration has been mainly followed in steady state conditions. For instance it was reported to increase several fold in correlation with the specific growth rate [32,33]. In response to stress ATP or the total adenylate concentration have been followed after cold shock, where both decrease [34], after UV radiation, where the ATP level transiently increased twofold [35], with a following decrease in *recA*^+ ^cells, which the authors relate to the action of substrate level phosphorylation. Also the levels of ATP and other nucleotides have been followed after induction of recombinant proteins by IPTG [36].

Studies with an appropriate short-time resolution of the ATP response in heat-shocked *E. coli *cells have not been published, although those data might be valuable to better understand the *in vivo *function of heat shock induced chaperones and proteases.

## Results

In response to a heat shock *E. coli *strongly induces the production of a number of proteins with protecting and degrading functions, which are ATP dependent. However, most of the data on protease and chaperone function were obtained by *in vitro *studies, and there are still nearly no data available indicating, that there is a sufficient level of ATP available after a heat shock. Rather confusing results from eukaryotes suggest, that the ATP level and the energy charge after heat shock quickly decrease, which could be seen as contradictory to the optimum conditions for the function of these proteins. Therefore it was the aim of this study to measure the response of the pool of adenosine nucleotides following a temperature up-shift in *E. coli*.

Aside from the measurement of the adenylate phosphorylation status we wanted to discuss the results in relation to the cellular metabolism and respiratory activity. Therefore the experiments were performed in a bioreactor, which allowed the measurement of oxygen consumption and carbon dioxide formation, as well as a fast sampling of nucleotides and medium samples.

All cultivations were performed with the *E. coli *K-12 strain W3110, which was a major strain in the evaluation of the *E. coli *stress responses. As indicated in the materials and methods section the cultivations were carried out on mineral salt medium with glucose as carbon source to avoid complex reactions in view of amino acid availability. The medium used was originally designed for high cell density fermentations, and therefore insures, that all salts are maintained at appropriate concentrations up to the end of the experiment.

**Figure 1:**
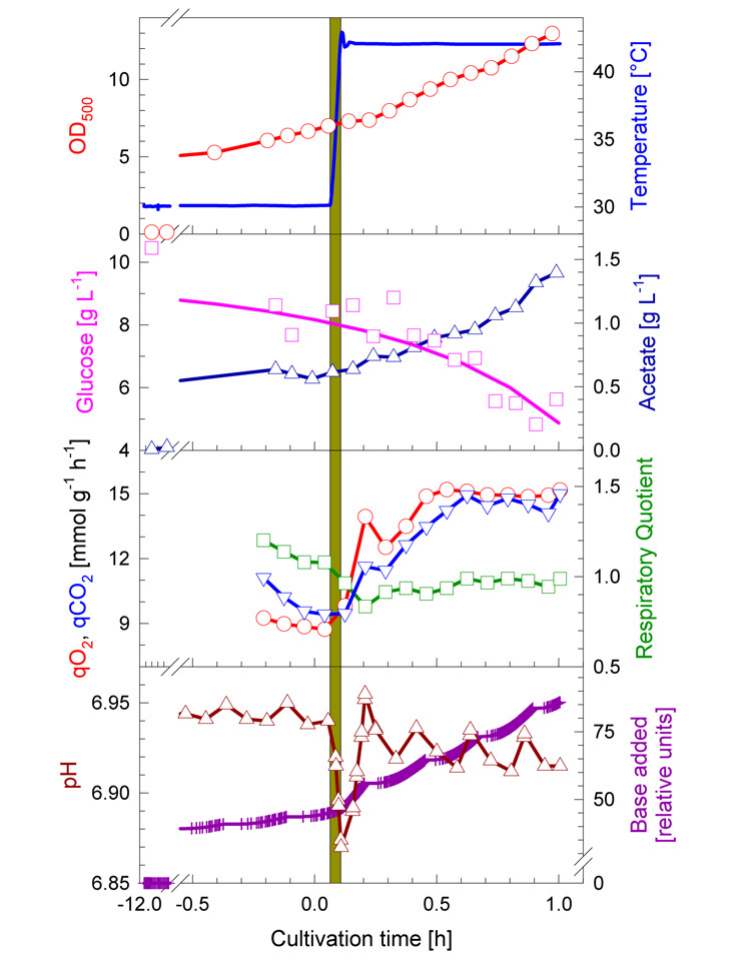
Batch cultivation of *E. coli *W3110 with a temperature up-shift performed at time 0 by switching the temperature set-point from 30 to 42°C. (A) OD_500 _(○), temperature (—); (B) glucose (□), acetate (△); (C) q_O2 _(○), q_CO2 _(▽), RQ (□), (D) pH (△), relative units of ammonia added (+). The grey area indicates the time period of about 5 min during which the temperature increased.

The experiments were performed with a temperature shift from 30°C to 42°C. In this range the viability of the cell is not significantly affected, although the heat shock response is strongly induced. The basic growth behaviour and parameters are shown in Fig. 1 and Table 1, respectively. Cultivations started with a batch phase at 30°C for about 11 hours, corresponding to 6 generations, to ensure a steady state of cellular parameters. The specific growth rate μ was determined to be 0.35 h^-1^. By following the glucose and acetate concentrations (see Fig. 1) the specific rate for glucose uptake (q_S_) was determined to be 5.1 mmol g^-1 ^h^-1 ^and the specific acetate production rate (q_A_) was 2.5 mmol g^-1 ^h^-1^. The temperature up-shift to 42°C was performed at a cell density of OD_500 _= 3 (corresponding to a dry cell weight of 0.675 g L^-1^) within 3 min. After the temperature shift the cultivation was continued for one hour. Rapidly after the temperature shift the cells start to increase the specific growth rate to 0.70 h^-1 ^and correspondingly the rates for glucose uptake and acetate production (see Table 1).

**Figure 2:**
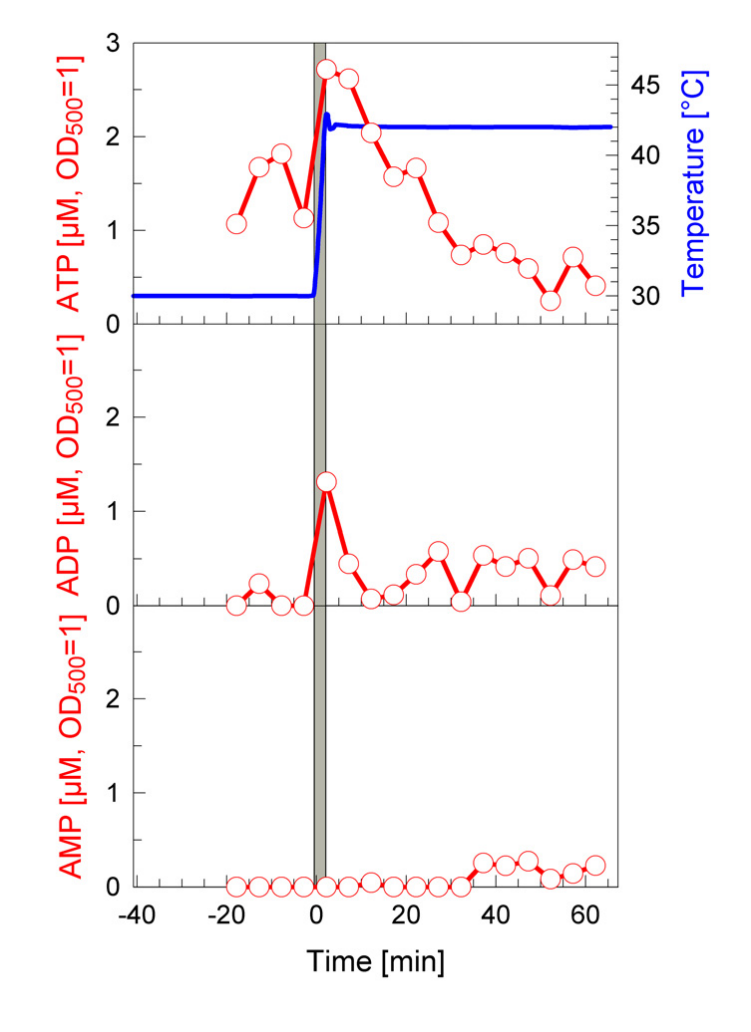
Response of the adenosine nucleotide pool to a temperature up-shift from 30 to 42°C in *E. coli *W3110. (A) ATP and cultivation temperature, (B) ADP, (C) AMP. The data were obtained during the batch cultivation shown in Figure 1.

**Figure 3:**
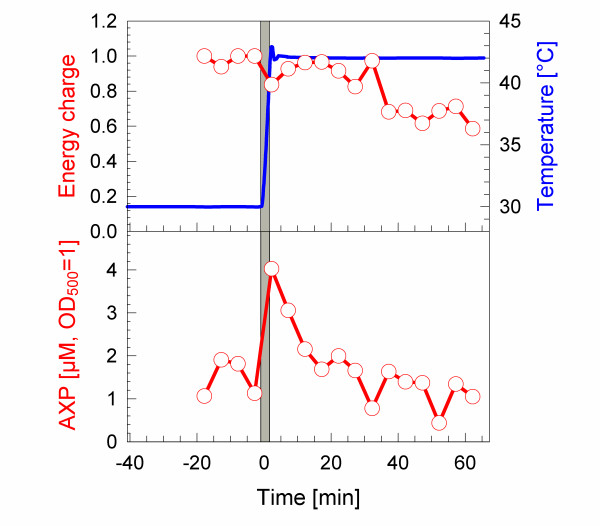
Dynamics of the Energy Charge and sum of adenosine phosphates (AXP) during a cultivation of *E. coli *W3110 with temperature up-shift from 30 to 42°C. Data were calculated from primary concentrations of the adenosine nucleotides shown in Figure 2.

During growth at 30°C the ATP concentration was detected to be about 1.5 μmol per OD_500_. Concentrations of the other lower phosphorylated adenosine nucleotides were very low during this phase and the resulting adenylate energy charge (EC) was above 0.95. Nucleotide samples were collected in five minute intervals. The ATP concentration increased to 2.75 μmol per OD_500 _immediately after the temperature shift to 42°C and also the ADP concentration was found to transiently increase significantly (see Fig. 2). After the transient increase the cellular concentrations of both nucleotides decreased and finally we detected a slight increase of AMP towards the end of the cultivation. Interestingly, the elevation of the ATP level was connected to a total increase of all adenosine phosphates, resulting in a slightly lowered EC to 0.8 (see Fig. 3). However, similar to ATP, also the sum of adenosine phosphates increased only transiently, approaching to the pre-shift level about 20 min after the temperature up-shift. The slight increase of AMP to the end of the cultivation influenced the energy charge, which decreased to 0.65. The transient increase of ATP after the temperature up-shift is connected to a high respiratory activity (Fig. 1C) and occurs in the phase, where also the flux of glucose is rapidly increasing.

In alternate series of experiments the temperature was shifted either from 37°C to 48°C, or from 35°C to 55°C which leads to growth inhibition and cell death. In both cases the temperature up-shift caused a strong transient increase of respiration and, at least in the first case we also found the rapid increase of ATP, however, the following reduction of the ATP level was much faster (data not shown).

**Figure 4:**
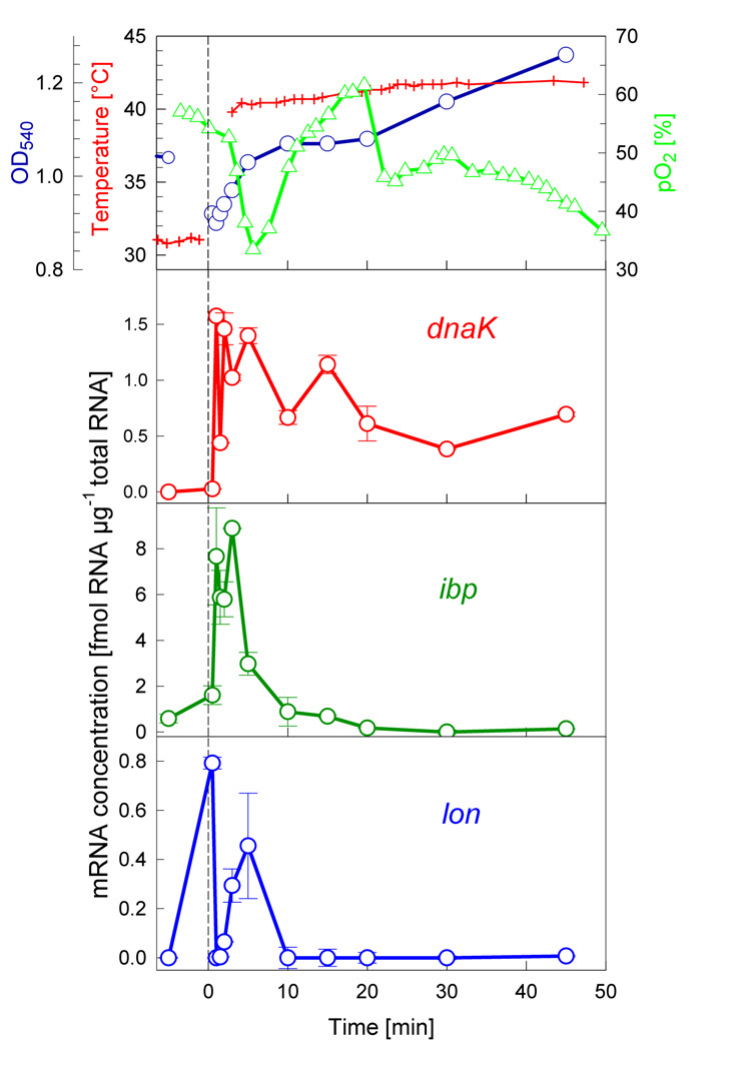
Response of mRNAs in a stirred-flask cultivation of *E. coli *W3110 with a temperature shock from 30°C to 42°C. For experimental details see Materials and Methods.

Further it was investigated how the time kinetics of the adenosine nucleotides are related to the expression profiles of typical heat shock genes. These experiments were performed in shake flasks with on-line monitoring of dissolved oxygen and temperature. A fast temperature up-shift was reached by diluting the culture with pre-heated medium which explains the decrease of OD_580 _in Fig. 4. Interestingly, the induction of the three investigated heat shock controlled genes was different. The expression of *dnaK *and *ibp *were both induced very rapidly and reached their maximum mRNA levels within only 1 min after the heat shock, but *lon *reached the maximum RNA level after 5 min. A quantitative evaluation of the mRNA amounts showed the highest concentration for *ibp*, but lower expression of *dnaK *and the lowest for *lon*. The level of *dnaK *mRNA was continuously significantly higher expressed than before the shift, but the mRNAs of *ibp *and *lon *were very low or not any more detectable after only 10 min. Interestingly, this shut-off of the expression of these genes is at about the same time when the pre-shift level of nucleotides was re-established.

## Discussion

Our studies indicate, that *E. coli *responds to a temperature up-shift by rapidly increasing respiration and glucose uptake. The increase of glucose uptake is possibly mainly directed to glycolysis and, consequently, to energy production. Concomitantly we detected a significant drop in the pH and an increase of the acetate level. The higher production of acetate per glucose (Y_A/S_) might point to an increase of glycolytic activity, and suggests that the flow to the tricarboxylic acid cycle was not increased correspondingly.

We detected a significant increase of the concentration of the sum of adenosine nucleotides (AXP) directly during the temperature up-shift which possibly is caused by RNA degradation as is concluded from the total RNA concentrations per cell which decreased to less than 50 % if the samples before and after the temperature up-shift are compared (data not shown). This increase of the AXP concentration was connected to a decrease in the energy charge, as the increase of ADP was higher than the increase of ATP. It remains to be investigated whether this relative transient increase of ADP has physiological effects. For example it might be hypothesized that the high concentration of ADP transiently inactivates various chaperones (see e.g.[37,38]) which bind ADP with higher affinity compared to ATP.

After the heat shock the cells strongly increase respiration and CO_2 _production which should be related to ain increased glycolytic flux, as correspondingly acetate formation is increased. A similar behaviour of increased glycolytic flux we have observed from other cultivations where a stress was caused by strong induction of a recombinant protein and also provoked a transient increase of the ATP level and higher respiration. However, differently from the investigated temperature up-shift, after induction of the recombinant protein the cells lost their viability [39]. Also a lethal heat shock caused by a temperature up-shift from 37°C to 52°C [40] or from 37°C to 48°C (own results, not shown) resulted in a transient increase of ATP, but this was much slower and the increase was only about 30 % with a maximum at about 30 min after the temperature shift.

Our data suggest that similarly as known for the heat shock response, that the response of the nucleotides is transient and the energy charge is restored 15 to 20 min after the heat shock. After this transient induction of the heat shock response cell growth recovers and it may be hypothesised that the cellular synthetic activities and metabolite pools reach a new steady state.

## Conclusion

This study shows that the heat shock response in *E. coli *in connection to the adenosine phosphates is different from the response of eukaryotes. Whereas different authors described in eukaryotes an immediate decrease of the ATP level following a temperature up-shift (e.g. [29-31]), in *E. coli *we found a transient increase, before the ATP level is lowered. It may be hypothesised, but has to be verified by further experiments that this transient increase of the ATP concentration is important for the action of chaperones and proteases and consequently for the function of the cellular system towards a heat shock response.

## Methods

### Strains

All experiments were performed with the *Escherichia coli *K-12 strain W3110 [F^-^, IN(*rrnD rrnE*)1, λ^-^] which was kindly obtained by the *E. coli *Stock Center (New Haven, USA).

### Cultivation medium and cultivation conditions

Phosphate buffered mineral salt medium according to Teich et al. [41] with 10 g L^-1 ^glucose and thiamine hydrochloride (0.1 g L^-1^) was used in all cultivations. The pH of the medium was set to 7.0 before sterilisation by addition of NaOH. For fermentation experiments two precultivations were carried out at the appropriate temperature of the main cultivation. The first culture was performed in 10 mL of nutrient broth (NBII: 1 % tryptone, 0.6 % yeast extract), whereas the second preculture contained 200 ml of the fermentation medium in a 1000 mL baffled Erlenmeyer flask. Each preculture was used at the exponential growth phase as inoculum for the next cultivation.

The fermentations were performed in a 6 L Biostat ED bioreactor (B. Braun Biotech International, Germany) with a starting volume of 4 L of mineral salt medium pH 7.0). The pH was controlled to drop not below 7.0 by addition of 25 % ammonia. Air flow (0.002 to 2 vvm) and stirrer speed (200 to 800 rpm) were increased stepwise to keep the dissolved oxygen tension above 20 %. After the initial batch phase to an optical cell density (OD_500_) of 3 the temperature up-shift was performed with a temperature increase from 30 to 42°C within 6 min.

Batch cultivations with on-line monitoring of temperature and oxygen were performed in 1000 mL baffled Erlenmeyer flasks with three side necks for the sensors and a needle for sampling into a syringe. The cultures were performed in 200 mL M9 mineral salt medium with 10 g L^-1 ^glucose, 2 mL L^-1 ^of trace element solution and 0.1 g L^-1 ^thiamine hydrochloride on a magnetic stirrer bar at 1000 rpm in a tempered water bath. The heat shock was performed by addition of 66 mL fresh M9 medium which was preincubated at 100°C and following replacement of the flask into another water bath (42°C). Temperature and oxygen were monitored by sensors (Pt1000, polarographic oxygen electrode from Meredos GmbH), connected to the wireless Senbit system (teleBITcom GmbH) with on-line data collection.

### Analytical methods

#### Cellular growth

Growth of the cultures was followed by measuring the optical density at 500 nm (OD_500_, for cultivation in the bioreactor) or 540 nm (OD_540_, for shake flasks), and by determination of the cell dry weight (CDW). Therefore 1.5 mL cell suspensions were centrifuged in pre-weighed 2 mL eppendorf tubes and washed once with 0.9 % (w/v) NaCl solution. After removal of the supernatant the samples were dried to constancy at 60°C for at least 24 h. Colony forming units were analysed by plating at least three dilutions and each of these at triple of the culture broth on nutrient bullion plates, which were incubated for one to three days at 37°C.

#### Glucose and acetate

Samples from the cultivation were collected by direct filtration from the reactor through a 0.2 μm disk filter. Afterwards the samples were stored at -20°C for further analysis. The analysis of glucose was performed with the hexokinase/glucose-6-phosphate-dehydrogenase method (Kit no. 139106, Roche Diagnostics GmbH, Germany) scaled down to 96-well plates with four parallels for each sample as described by Larsson and Törnkvist [42]. Acetate was analysed with the Kit no. 148261 (Roche Diagnostics GmbH, Germany) correspondingly.

#### Outgas analysis

The outgas oxygen and carbon dioxide concentrations were measured with an URAS10E (Hartmann & Braun GmbH, Frankfurt, Germany). The specific rates (q_O2_, q_CO2_) were calculated continuously over the fermentation by including fitted biomass values.

#### Analysis of adenosine nucleotides

The adenosine nucleotides were analyzed with an RP-HPLC method as described earlier in detail Meyer et al. [43].

#### Analysis of mRNAs

10 mL samples of cell broth were immediately chilled with 1350 μL cold ethanol/phenol (95:5, v/v, pre-cooled at -20°C). After centrifugation at 10 000 rpm (4°C, 5 min) the supernatant was carefully removed, the pellet was resuspended in 1 mL of RNA Later (Ambion) and the samples were stored in 250 μL fractions at -70°C until analysis.

Total RNA extraction was performed with a total RNA isolation kit (A&A Biotechnology, Gdynia, Poland) according to the instructions of the manufacturer. Total RNA was quantified with the RiboGreen Quantification kit (Molecular probes). Quantitative analysis of *dnaK*, *ibp *and *lon *mRNAs was performed with a sandwich system earlier described in detail by Rautio et al. [44]. Therefore probes shown in Table 2 were designed using the CloneManager5 program with following submission of the sequences a NCBI BLAST search  to exclude alignments with other *E. coli *genes.

HPLC-purified unlabelled and biotin-labelled oligonucleotide capture probes were purchased from Metabion GmbH (Martinsried, Germany). Dig-tail labelling of the detection probes was performed according to manufacturer's instructions using the Roche Dig-tailing kit (2^nd ^generation, Roche Diagnostics GmbH, Mannheim, Germany).

*In vitro *RNA standards were designed for the quantitative analysis of each gene. Therefore primers as indicated in Table 3 were used for *in vitro *transcription (purchased from Sigma-Genosys, Cambridge, UK). Left primers (Primers 1) contained the 25 nucleotide T7 promoter sequence at the 5' side. Target genes were amplified by PCR from total DNA extracts of *E. coli*. The PCR products were purified using QIAquick PCR purification Kit and then used as templates for *in vitro *transcription using the MAXIscript T7 transcription kit from Ambion (Austin Texas, U.S.A.). The *in vitro *standards were quantified by the RiboGreen RNA Quantification Kit (Molecular probes) and a standard calibration curve with the respective standard was performed at the same plate where the samples were analysed.

Streptavidin MagneSphere Paramagnetic Particles (1 mg mL^-1 ^magnetic particles in PBS, 1 mg mL^-1 ^BSA and 0.02 % NaAzide) from Promega (Madison, USA) were used as magnetic beads for immobilisation of the target. Sandwich hybridisation was performed in 96-well plates at 50°C for 30 min with constant shaking (700 rpm). Therefore hybridisation solution with a final concentration of 5 × SSC, 20% formamide, 2% Denhardt reagent, 3% dextran sulphate and 0.2% SDS was added to a final volume of 100 μL to each sample. Each well of the plate contained 1 pmol of DIG-tail labelled detection probe, 5 pmol of biotin-labelled capture probe and appropriate dilutions of *in vitro *transcripts for the standard curve or of the RNA extracts from cultivation samples.

Immobilisation of RNA molecules was done by adding 20 μl streptavidin coated magnetic beads to each well after hybridisation. DIG labelled probes were bound to Anti-DIG-alkaline phosphatase (added 1:2000 in TBS-buffer) and the enzymatic reaction was carried out with AttoPhos (Promega) as substrate. Fluorescence was measured with the Wallac 2 fluorescence reader (Perkin Elmer Life Sciences) at 25°C using the Attophos1-program.

## Authors' contributions

SW and PN carried out the fermentations and nucleotide analyses. JS, CF, CM and DB developed the method for analysing the mRNAs and performed the corresponding analysis. JP and AV were included in the wireless analysis with the Senbit system. JS, PN and AV drafted the manuscript. All authors read and approved the final manuscript.

## Figures and Tables

**Table 1:** Cultivation parameters of parameters from batch cultivations of *E. coli *W3110 at 30°C and after a temperature shift to 42°C. The data are average values from two independent fermentations.

Parameter	before temperature shift	after temperature shift
μ [h^-1^]	0.38	0.71
q_S _[mmol g^-1 ^h^-1^]	5.1	17.4
Y_X/S _[g mol ^-1^]	75.6	41.4
q_A _[mmol g^-1 ^h^-1^]	2.5	8.0
Y_A/S _[mol mol^-1^]	0.30	0.51
Y_CO2/S _[mol mol^-1^]	1.86	0.80
Y_O/S _[mol mol^-1^]	1.76	0.85

**Table 2:** Probes used in Sandwich hybridization Capture probes were biotin labelled and detection probes contained a Dig tail.

***Name***	***Sequence (5'-3')***	***Position***
**dnaK**		
Helper probe 1	GTCGGGATAGTGGTGTTTTT	1259c
Capture probe	AGAACACCTGGCTGTGCTTG	1279c
Helper probe 2	CTGGTTGTCTTCAGCGGTAG	1299c
Detection probe	TTGTCGCCTGCTTCTTCAAC	1667c
**Ibp**		
Capture probe	ACCAGCCACAGCGATAGCAA	165c (ibpA)
Detection probe	TTCCAGTTCGCTCTCAGCAA	186c (ibpA)
Helper probe	ACCACCAGCAGATTATCCTG	215c (ibpA)
**Lon**		
Capture probe	ACTCCAGATACTCCGCCTTC	355c
Detection probe	GCCTGAATGGACTCCTGCAT	1919c

**Table 3:** Primers for synthesis of *in vitro *RNA standards. The T7 promoter sequence was added to the 5' side of all primer 1 sequences.

***Name***	***Sequence (5'-3')***	***Position***
**dnaK**		
Primer 1	CTCTTGTGTAGCGATTATGG	39
Primer 2	GTTGTTTGCAGAAGCATCAG	1860c
**Ibp**		
Primer 1	CCGCTTTACCGTTCTGCTA	22 (ibpA)
Primer 2	CTATTTAACGCGGGACGTTC	425c (ibpB)
**Lon**		
Primer 1	ATGAATCCTGAGCGTTCTGA	1
Primer 2	CACAACCTGCATACCAGAC	2342c
		
**T7 promoter**	CTAATACGACTCACTATAGGGAGA	
